# 16S rRNA Next Generation Sequencing Analysis Shows Bacteria in Alzheimer’s Post-Mortem Brain

**DOI:** 10.3389/fnagi.2017.00195

**Published:** 2017-06-20

**Authors:** David C. Emery, Deborah K. Shoemark, Tom E. Batstone, Christy M. Waterfall, Jane A. Coghill, Tanya L. Cerajewska, Maria Davies, Nicola X. West, Shelley J. Allen

**Affiliations:** ^1^School of Clinical Sciences, Faculty of Health Sciences, University of BristolBristol, United Kingdom; ^2^School of Biochemistry, University WalkBristol, United Kingdom; ^3^School of Biological Sciences, Life Sciences, University of BristolBristol, United Kingdom; ^4^School of Oral and Dental SciencesBristol, United Kingdom

**Keywords:** Alzheimer’s disease (AD), bacteria, human microbiome, 16S rRNA, next generation sequencing (NGS)

## Abstract

The neurological deterioration associated with Alzheimer’s disease (AD), involving accumulation of amyloid-beta peptides and neurofibrillary tangles, is associated with evident neuroinflammation. This is now seen to be a significant contributor to pathology. Recently the tenet of the privileged status of the brain, regarding microbial compromise, has been questioned, particularly in terms of neurodegenerative diseases. It is now being considered that microbiological incursion into the central nervous system could be either an initiator or significant contributor to these. This is a novel study using 16S ribosomal gene-specific Next generation sequencing (NGS) of extracted brain tissue. A comparison was made of the bacterial species content of both frozen and formaldehyde fixed sections of a small cohort of Alzheimer-affected cases with those of cognitively unimpaired (normal). Our findings suggest an increase in bacterial populations in Alzheimer brain tissue compared with normal.

## Introduction

Pathological triggers, culminating in the eventual loss of cognitive function in Alzheimer’s disease (AD), are widely acknowledged to occur up to two decades before symptoms arise (Bateman et al., 2012). It is acknowledged that the increased level of amyloid Aβ42 in the brain parenchyma, due to either increased production of amyloid or its decreased removal, is likely to contribute substantially to this. However, understanding why the presence of excessive levels of Aβ do not necessarily result in cognitive impairment (Katzman et al., 1988; Hulette et al., 1998; Price and Morris, 1999; Aizenstein et al., 2008; Esparza et al., 2013) may be related to the known role of inflammation and the importance of the response of the innate immune system, which are also recognized as essential factors (Heneka et al., 2015b). The common sporadic form of AD arises from a large number of possible risk factors. The presence of the E4 polymorphism of apolipoprotein E4 (*APOE*4) has long been known to be the most potent risk factor for sporadic AD, second only to age. One reason for this is likely to be its importance in the clearance of Aβ, another may be its influence on inflammatory response and its adverse influence on the integrity of the blood-brain barrier (BBB; Bell, 2012), which is pertinent when discussing the level of privilege the brain retains (Yu et al., 2014). The E4 polymorphism is proinflammatory, unlike the more common E3 form, which facilitates suppression of inflammation (LaDu et al., 2001; Guo et al., 2004; Chen et al., 2005). Further to this, multicenter genome-wide association studies (GWAS) have identified susceptibility loci on genes which may increase or decrease the risk of AD (Bertram and Tanzi, 2009). The polymorphisms found by these studies to be associated with AD are thought to mainly affect three functional systems: immune and inflammation responses, lipid metabolism and endosomal vesicle recycling (Tosto and Reitz, 2013; Guerreiro and Hardy, 2014). Evidence suggests that the influence of neuroinflammation is involved at an early stage of AD (Akiyama et al., 2000b; Holmes et al., 2009; Perry et al., 2010; Perry and Holmes, 2014) and it has been demonstrated that a microbiological insult, including bacteria or virus, may trigger, or contribute to neuroinflammation and subsequent neurological damage (Miklossy, 2011; Mawanda and Wallace, 2013; Hill et al., 2014; Cerajewska et al., 2015; Olsen and Singhrao, 2015, 2016; Shoemark and Allen, 2015; Itzhaki et al., 2016; Miklossy and McGeer, 2016).

The evidence so far is reliant on histology and other methods that require prior knowledge of which bacterial species to look for. Here we use 16S ribosomal RNA gene next generation sequencing (NGS) in a pilot comparative study in normal and AD-affected brains to determine the range and extent of bacterial species present in this brain tissue.

## Materials and Methods

### Cohort Study

Frozen and paraffin embedded tissue was obtained, with local Research Ethics Committee approval, from the South West Dementia Brain bank (SWDBB), University of Bristol, UK. (SWDBB #ITA058). All studies conformed to relevant regulatory standards. All brain samples are routinely assessed by the South West BB for prion disease; only brain samples from subjects free of prion disease pathology were released for this study. Handling of the samples required the use of suitable personal protective equipment including mask and protective eye-wear, and was carried out in a lamina flow hood, to prevent contamination in either direction. Left hemispheres are routinely sliced and frozen at −80°C; the right hemispheres are formalin fixed for neuropathological assessment and for immunohistochemical analysis. For this study, formalin fixed paraffin embedded sections (denoted here as S) and tissue from frozen slices (denoted here as F) were used from the temporal cortex (BA 38/40) of patients with AD and non-demented controls (C). The diagnosis of AD was according to standard criteria as specified in the Diagnostic and Statistical Manual of Mental Disorders Fourth Edition (DSM-IV; American Psychiatric Association, 2000). Table 1 shows the properties of the cohort. Control and AD samples were then age and post mortem delay (PMD) matched except for an additional control (#678) which was added to allow assessment of changes of bacterial content with extended PMD (216 h).

**Table 1:** Information on cases studied.

Control (*n* = 12)				AD (*n* = 14)				
BB No.	Age (years)	Sex M/F	PMD	BB No.	Age (years)	Sex M/F	PMD	Braak Stage
3	78	M	48	7	73	F	11	n.d.
69	64	M	16	148	85	F	24	n.d.
90	78	M	12	172	77	F	23	n.d.
102	70	M	50	251	74	F	11	5
294	65	M	9	435	54	F	24	6
295	82	M	3	498	83	F	5	5
412	82	F	96	508	80	M	5	5
461	77	M	10	584	85	F	85	6
467	75	M	6	598	56	F	44	6
678	84	F	(216)	684	81	M	4	6
721	90	M	5.5	713	62	M	24.5	6
781	87	M	24	718	98	F	21.25	5
				772	86	F	73	5
				885	88	F	7.5	6

*Brain bank (BB) no. is number assigned to a brain by South West Dementia Brain Bank (SWDBB), post mortem delay (PMD) is time interval (delay) between death and post-mortem; Braak stage refers to neurofibrillary tangle stage where 6 is the most advanced stage of dementia, both stages 5 and 6 have extensive neurofibrillary deposits; n.d. is not determined. Samples taken for further next generation sequencing (NGS) are shown in Table 2*.

### DNA Extraction

DNA was extracted from frozen tissue, first by homogenization of 50–100 mg using a pellet pestle (Sigma) in a 1.5 ml microcentrifuge tube. The homogenate was resuspended in 1ml of Tris EDTA (T.E.) buffer (10 mM Tris pH 8.0, 1 mM EDTA), extracted with 0.5 ml of phenol/chloroform/isoamyl alcohol (Sigma), then 0.5 ml chloroform, and DNA ethanol precipitated with 2 volumes of ethanol in the presence of 0.2 M NaCl. After sedimentation at 16,000× *g*, the DNA pellet was washed with 70% then 100% ethanol and air-dried before being dissolved in 50 μl of T.E. buffer. The entire procedure was carried out under sterile conditions in a lamina-flow hood. DNA from formalin-fixed, paraffin-embedded (FFPE) sections was extracted using the Qiagen DNA FFPE Tissue kit (Qiagen 56404) according to the manufacturer’s instructions except for the following modifications: the outermost 2 mm of each section was removed using a 1 ml sterile pipette tip along with all surrounding paraffin. The section was then incubated at 85°C for 1 h to melt the paraffin, which was then removed by incubation for 10 min in xylene. Each section was washed *in situ* extensively with 100% ethanol using a wash bottle. The tissue was then scraped into a 1.5 ml centrifuge tube as stipulated in the protocol. The area from which the tissue was removed was then washed with 180 μl of ATL tissue lysis buffer (Qiagen) which was pooled with the tissue. From this point onwards the method was according to the manufacturer’s protocol.

### DNA Quantification

Initial DNA concentrations were obtained by A_260/280_ absorption using a NanoPhotometer P-Class (Implen, Munchen, Germany). Most samples gave an A_260/280_ ratio between 2 and 1.8. Samples with ratios lower than 1.7 were rejected.

### PCR Primer Design

The primary aim of this study was to assess the presence in the brain of bacteria from the widest possible taxonomical spectrum. Therefore, universal bacterial 16S rRNA PCR primers were chosen for maximal taxonomical coverage. In order to achieve this, representative 16S ribosomal gene sequences from the major phyla commonly found in the human microbiome, Actinobacteria, Bacteroidetes, Firmicutes, Fusobacteria and Proteobacteria, obtained from the National Center for Biotechnology Information (NCBI) 16S ribosomal RNA database, including representatives of the major human pathogens (Chakravorty et al., 2007) and oral microbiome (Dewhirst et al., 2010) were aligned using Clustal Ω (EMBL-EBI, Wellcome Genome Campus, Hinxton, Cambridgeshire). The universal variable region-3 primer F342 (5′-CCTACGGGAGGCAGCAG) was derived and used in combination with the reverse primer 518R (5′-ATTACCGCGGCTGCTGG). These primers are designated “primer pair 1”. They are similar to those described by Chakravorty et al. (2007) who systematically assessed 16S variable regions for their ability to distinguish between 110 bacteria, representing a wide spectrum at the genus level, and tested with a mixed population containing 24 different bacterial genera. Dendrogram analysis showed that this primer pair could distinguish between all 110 species examined. Mori et al. (2014) also carried out a systematic study of possible universal 16S PCR primers that had low probability of amplifying eukaryotic sequences. Apart from one G to A substitution, their primer 342F is the same as that described here and showed good taxonomic coverage.

### PCR

Each amplicon was generated using 700 ng of starting material in a 50 μl reaction containing 1*×* Platinum Taq buffer with 0.2 μl Platinum Taq (ThermoFisher Scientific, Waltham, MA, USA), 1.5 mM MgCl_2_, each nucleoside triphosphate (NTP) at 200 μM and each primer at 1 μM final concentration. An initial 5 min denaturation step at 95°C was followed by 40 cycles of 95°C, 30 s; 65°C, 30 s; 72°C, 30 s with a final 7 min extension at 72°C.

### PCR Analysis

The PCR of the variable region 3 was repeated using 1200 ng of starting material on an extended, but overlapping cohort of frozen samples. Consistent with the original PCR, the amplicon consisted of two bands (bands 1 and 2, Supplementary Figure S1) superimposed over a faint smear. Band 1 is approximately 200 bp, which corresponds to the variable region-3 product of the majority of bacterial species using these primers. The smaller band 2 is consistent with the product size predicted for both the human 18S product (174 bp) and Propionobacteria and Corynebacteria (168 bp).

### Amplicon Processing

Amplicons were electrophoresed in a 2% agarose gel using 1*×* Tris-acetate-EDTA buffer (T.E.A. buffer: 40 mM Tris pH 7.6, 20 mM acetic acid, 1 mM EDTA) and purified by Qiaquick Gel Extraction kit (Qiagen GmbH, Hilden. Germany#28704). Amplicons were further purified using the Agencourt Ampure XP beads (Auto Q Biosciences Ltd, UK) and then quantified using the High Sensitivity Qubit kit (ThermoFisher Scientific). Amplicon sizes were determined using the DNA 1000 Tapestation assay (Agilent Technologies, US). Using the amplicon size and Qubit concentrations, the sample concentrations were normalized to 10 nM. A pool of amplicons at 10 nM was created by adding 5 μl of each normalized amplicon to a single pool. The pool was re-quantified using the Qubit High Sensitivity assay to determine the volume required to take 100 ng into the library preparation stages. The concentration of the amplicon pool was 1.6 ng/μl.

### Library Preparation

One-hundred nanogram of the amplicon pool was taken into the Life Technologies Ion Plus Fragment Library Kit (ThermoFisher Scientific) protocol for amplicons without fragmentation and the protocol was followed without deviation. The resulting library, created by IonXpress Adapter 5 (ThermoFisher Scientific; sequence CAGAAGGAAC), was verified for size using a TapeStation High Sensitivity DNA 1000 assay (Agilent Technologies, US).

### Sequencing

Template generation and sequencing were performed using the following Life Technologies kits: Ion PGM OT2 400 Kit; Ion PGM Sequencing Kit 400; Ion 318v2 chip. Protocols were followed according to manufacturer’s instructions without deviation.

### NGS Analysis

NGS data was processed using a custom quality control (QC) pipeline including the use of seqtk and trimmomatic (Bolger et al., 2014) which de-convoluted and trimmed the barcode sequences from the reads and filtered for quality by minimum and maximum sequence length. Resulting reads were then processed using Qiime^1^ (Caporaso et al., 2010b). Sequence chimera filtering was carried out using both reference and *de novo* methods, with only sequences that pass both these being retained and operational taxonomic units (OTUs) selected based on a 97% similarity with a minimum of 3 reads representing each OTU, both methods utilizing uclust (Edgar, 2010; Edgar et al., 2011). Following alignment to the Greengenes Core reference alignment (DeSantis et al., 2006) using PyNAST (Caporaso et al., 2010a), taxonomies were assigned using the uclust method (Edgar, 2010; Edgar et al., 2011) and phylogeny generated using FastTree (Price et al., 2010). An OTU table was generated from these results, and alpha and beta diversity metrics generated using the standard QIIME tools, with the latter being calculated using UniFrac (Lozupone and Knight, 2005) and visualized using Emperor (Vázquez-Baeza et al., 2013). Data summarized in excel format is supplied as Supplementary Data. Original data is available at https://drive.google.com/drive/folders/0B5cL36CHc9tyMkZqVGZPeXFwUkk

## Results

### Amplicon Generation and Assessment of Possible Contamination

Using primer pair 1, amplicons were generated that contained bands ranging approximately from 170–220 bp. These were gel purified and concentrations normalized prior to library preparation and NGS analysis.

To assess taxonomic coverage, observed taxonomic diversity was plotted against sampled read depth using alpha rarefaction analysis. This showed that a read depth of 20,000 was required for adequate representation of OTU diversity. Control samples F781C, F102C, F90C, F721C and S412C fell below this threshold and, therefore, the data from these may not fully represent the taxonomic diversity of the bacterial populations from these samples. The samples analyzed by NGS are described in Table 1. Age ranged from 62 years to 98 years, with Braak stages of 3 or 4 (controls) and 5 or 6 (AD).

It was not possible to completely avoid peri- or post-mortem contamination and, in the case of FFPE sections, contamination during storage (in cardboard holders). It was for these reasons that both types of sample were analyzed in parallel; 102C and 781C were included as both FFPE and frozen samples. Sample 781C was relatively consistent between the different sources, with both FFPE section (S781C) and frozen tissue (F781C) bacteria comprising approximately 40%–45% Actinobacteria and 35%–45% Proteobacteria with 10%–15% Firmicutes and 2% other. FFPE sections from 102C contained predominantly Proteobacteria, whereas the frozen tissue sample of 102C (F102C) was predominantly composed of Fusobacteria. This increased Fusobacteria contribution was almost entirely confined to this one sample. Therefore, these data suggest that, although there is variability, neither type of sample preparation introduced method-specific bias or contamination.

**Figure 1:**
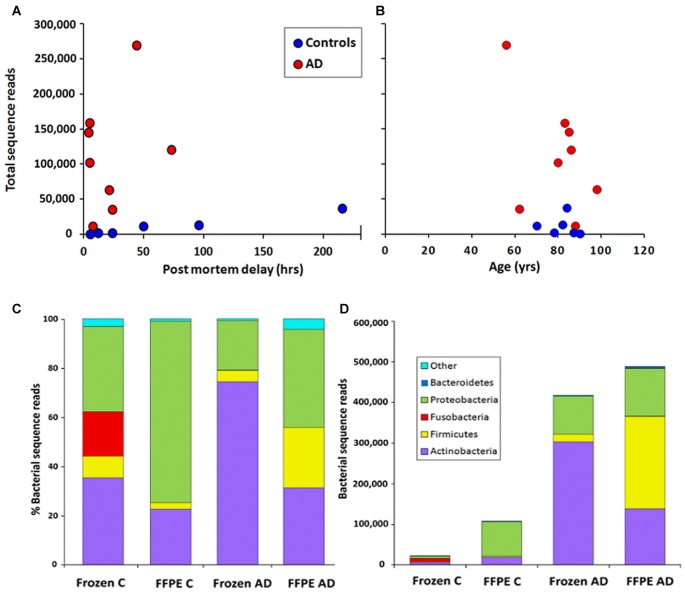
Correlation of total bacterial reads with **(A)** post-mortem delay (PMD; in hours) and **(B)** age (in years) *n* = 6 C; *n* = 8 Alzheimer’s disease (AD). **(C,D)** Phylum level comparison of bacterial populations in frozen and formalin-fixed, paraffin-embedded (FFPE) samples from control **(C)** *n* = 8; AD *n* = 8. **(C)** shows percentage composition **(D)** shows bacterial sequence reads.

In order to assess levels of peri-mortem contamination, PMD was plotted against total bacterial reads for each individual (Figure 1A). Neither bacterial reads from AD nor control correlated with age by linear regression analysis (*p* = 0.80 and 0.11 respectively). PMD did not significantly correlate with bacterial reads in control (*p* = 0.055) or AD (*p* = 0.491). The sample number is too low for definitive analysis, but these data suggest that contamination from PMD is not a significant factor and, furthermore, increased levels of bacterial reads are associated strongly with AD compared to normal individuals and not with age (Figure 1B).

**Figure 2:**
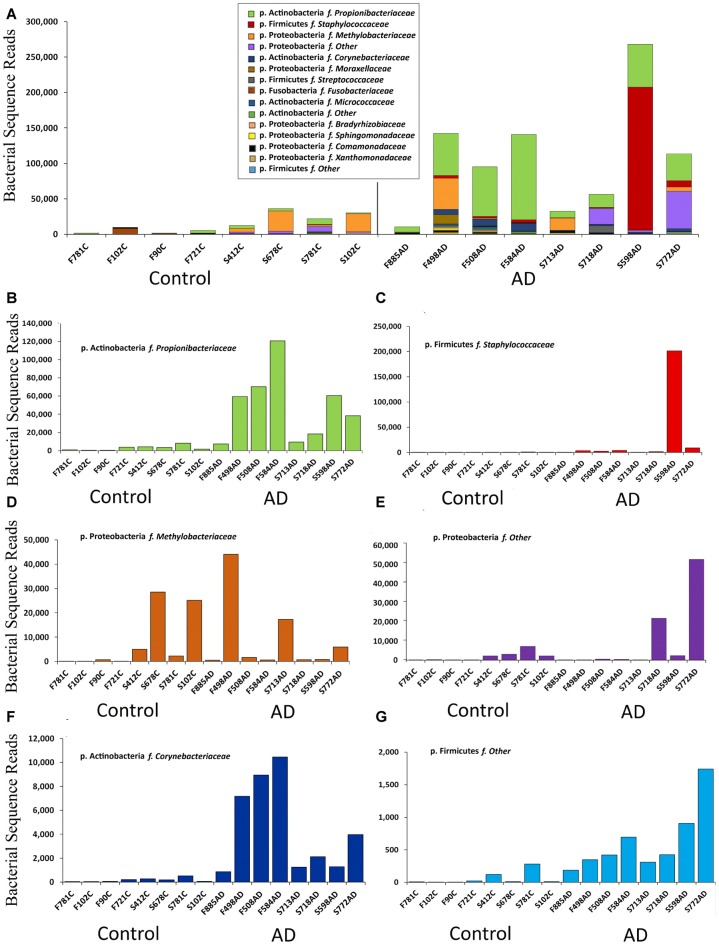
Summary of data at the family level expressed in bacterial sequence read counts. **(A)** Bacterial reads in control (*n* = 8) and AD (*n* = 8) samples. Legend is given in same order as shown in the bars (p. is phylum, f. is family). **(B–G)** Selected individual bacteria representing major components at the family level with p. Firmicutes f. *Staphylococcaceae*; p. Actinobacteria f. *Corynebacteriaceae*, showing higher levels in AD but p. Proteobacteria f. *Methylobacteriaceae* distributed across both AD and control samples.

Contaminating exogenous DNA also provides a major technical difficulty. This is especially true of low biomass experiments and where target DNA is extremely dilute (Lusk, 2014; Salter et al., 2014). Ideally, “no template” controls should be included. These controls consistently identified multiple Alpha-proteobacteria including Methylobacteriaceae; multiple Beta-proteobacteria, Gamma-Proteobacteria, including Enterobacteriaceae and Escherichia, Firmicutes including Streptococcus (but not Staphylococcus), Actinobacteria including *Corynebacteriaceae* and *Propionibacteriaceae*, Bacteroidetes, Deinococcus and Acidobacteria (Salter et al., 2014; Salzberg et al., 2016). These contaminants vary between repeat experiments, are laboratory and operator-specific and can derive from any stage in the metagenomics sequencing process, including DNA-extraction kits and molecular biology-grade water (Salter et al., 2014). In the study presented here, although a PCR product was invariably generated in no-template controls, yields were too low to analyze by NGS without further amplification and indeed, control brain samples did not generate enough amplicon to produce fully representative libraries. AD and control samples were treated in exactly the same manner, yet massively more bacterial 16S reads were yielded from AD samples, strongly suggesting that contamination is not a major issue for these data. One possible source of bacterial “contamination” in our tissue, blood, needs to be addressed. A recent 16S NGS study carried out on normal (control) blood samples provides a good base-line for this (Paisse et al., 2016). Blood is shown to contain around 1.8–7.6 × 10^7^ 16S sequences per ml of whole blood. These levels must be reflected in our data to some extent. The taxonomic profile seen in our study for non-AD brain is similar to that for blood as shown by Paisse et al. (2016; Table 3) with Proteobacteria by far the highest percentage. In comparison, our profile of AD brain is different with Actinobacteria as the largest component. Additionally, for these data, the largest Proteobacteria component is Alpha-proteobacteria; Rhizobiales; *Methylobacteriaceae* (Figure 2D), which as an environmental rhizobial bacterium is listed as a common contaminant (Laurence et al., 2014) and displays a random distribution between control and AD brains (Figure 2D).

**Table 2:** A summary of NGS data.

Summary of NGS data		Control			AD		
		Sample	Total reads	Bacterial reads	Sample	Total reads	Bacterial reads
Number of samples	16	**F781C**	2473	2074	**S713AD**	43,237	35,510
Number of observations	2575	**F90C**	3655	2291	**F885AD**	49,733	11,745
Total counts	1,933,972	**F721C**	8458	6243	**S718AD**	66,180	63,365
Counts per sample (min)	2473	**F102C**	11,557	11,481	**S772AD**	122,273	120,282
Counts per sample (max)	685,992	**S412C**	15,233	13,385	**F584AD**	163,267	145,295
Median	41,126.5	**S781C**	25,269	24692	**S598AD**	291,581	269,504
Mean	120,873	**S102C**	32,552	31500	**F508AD**	373,496	102,261
Standard Deviation	179,469	**S678C**	39,016	37275	**F498AD**	685,992	158,506

* Brain bank numbers have a prefix of F or S, representing samples from frozen tissue or from a section respectively. A suffix of C or AD denotes a sample from control or AD brain, respectively. Controls (*n* = 6; two were present as both formalin-fixed, paraffin-embedded (FFPE) and frozen), mean ± standard deviation: age: 81.8 ± 7.1 years, PMD: 37.5 ± 36.9 h (excluding S678C which has a PMD of 216 h) or 67.3 ± 79.9 h (including S678). AD (*n* = 8), mean ± standard deviation: age: 79.8 ± 13.9 years, PMD 33.2 ± 31.3 h*.

**Table 3:** Comparison with briefly summarized approximate data at phylum level from the closest comparable studies (Branton et al., 2013; Paisse et al., 2016).

Study	Present	Study	Branton et al. (2013)	Paisse et al. (2016)
**Sequencing Method**	NGS 16S v3	NGS 16S v3	Deep Sequencing of cDNA	NGS 16S v3/v4
**Tissue**	AD (*n* = 8)	Control (*n* = 6)	ODC PM Brain	Blood BC
			HIV PM Brain	Blood Plasma
			Surgical	Blood RBC
**Average age (years)**	79.8	81.8	ODC 62	21
			HIV 38	
			Surgery 24	
**Proteobacteria**	105,382	43,986	51.8*	3.2–3.7 × 10^7^**
	(30%)	(54.5%)	(65–80%)	(80–87%)
Sections/Frozen%	(40/20)%	(74/35)%		
**Actinobacteria**	221,001	13,261	8.8*	2.8–4.2 × 10^6^**
	(52.5%)	(28.75%)	(5–17%)	(7–10%)
Sections/Frozen%	(31/74)%	(22/35)%		
**Bacteroides**	2,520	621		1 × 10^6^ **
	(0.9%)	(1.25%)	(0–35%)	(2–3%)
**Firmicutes**	122,732	2,186	Mostly not seen	1.3 × 10^6^ **
	(15%)	(6%)		(3–6%)

The first two columns show AD and control bacterial reads with percentage reads in brackets. % from sections and frozen tissue are given in brackets below this for Proteobacteria and Actinobacteria. Other disease controls (ODC) *Average (*n* = 10) **Average whole blood data based on an average of 4.2 × 10^7^ total bacterial reads per ml of whole blood. BC is buffy coat; RBC is red blood cell. HIV is human immunodeficiency virus; PM is post-mortem.

### Primer Specificity and Taxonomic Coverage

Primer pair 1 generated an NGS data set containing 23 phyla and 178 taxa at the family level. However, 342F has 86% and 518R 100% identity to equivalent sites within the human 18S rRNA gene and together they generate a 168 bp human 18S PCR product with high efficiency. Therefore, the human 18S product was co-purified along with the bacterial amplicon. This resulted in the largest operating taxonomic units being taxonomically unassigned in the initial analysis with subsequent BLASTN analysis of representative sequences against the NCBI nucleotide collection revealing them to be either an uncultured bacterial species (accession KJ766015.2; 252/252 query coverage: 100%, E value: 1e-63, identity: 100%) or, using Clustal Omega alignment, human 18S rRNA (NR046235.1). These data could not differentiate between these two candidates but it is thought, in all probability, to be the latter. Unassigned OTUs, all of which were h18S, were subtracted from the total read counts for each individual to give revised total bacterial reads (Table 2) which were used in all subsequent calculations of percentage composition (Table 3).

Two individuals, both AD (498, 508) had large numbers of human 18S reads. This remains unexplained; but could be due to inconsistency in gel purification or amounts of human genomic DNA present in each sample.

### Comparison of Bacterial Populations in AD and Control Samples

This NGS study was carried out with normalized, re-amplified libraries and therefore was not designed to assess actual bacterial numbers. However we see here a clear pattern with AD samples yielding noticeably more bacterial reads than controls. This is depicted in Figures 1D and 2.

Figure 1C shows the percentage compositions of each of the four groups (FFPE controls, FFPE AD, Frozen Control and Frozen AD) at the phylum level. Figure 1D which shows average total bacterial reads for each of the four groups, showing that there is a 5–10-fold more bacterial reads in AD compared with control in both frozen tissue and FFPE sections.

Table 3 compares these data with two other relevant studies: massively parallel sequencing of cDNA generated from total RNA from surgically removed brain samples and post-mortem material, yielding total human cDNA sequences as well as microbial sequences (Branton et al., 2013) and specifically 16S rRNA-directed-NGS on blood (Paisse et al., 2016). These two studies and ours produce data in broad agreement with each other with the same four phyla contributing between 81%–100%. Branton et al. (2013) differs somewhat from the rest with large variations in Bacteroides up to 35% compared to 0.3%–3%. Our data differs from the other two in Actinobacteria content with between 22%–74% compared to 5%–17% and Proteobacteria with 20%–74% compared to 65%–87%. These data also differ from the others in the composition of the Proteobacteria component, with three out of four groups having a majority of Alpha-proteobacteria with only one group having Beta-proteobacteria as the predominant class.

Notably our data suggests Actinobacteria reads are higher in AD samples compared to controls, with Proteobacteria having a roughly inverse relationship. Firmicutes has a greater percentage of reads in AD in FFPE, but not in frozen tissue, and this was due to a large staphylococcal presence in one sample. Actinobacteria are somewhat higher in our controls compared to Branton et al. (2013) and Paisse et al. (2016) and consistently higher in AD samples (Table 3 and Figures 2B,F). The Actinobacteria content seen here consists primarily of *Propionibacteriaceae*, (Figure 2B) with the largest OTUs being *P. acnes*. Actinobacteria accounts for up to 10% of total reads in blood (Paisse et al., 2016) and includes *Corynebacteriaceae* and 10 other Actinobacteria taxa but no *Propionibacteriaceae*. *Corynebacteriaceae* (closely related to *Propionibacteriaceae*) constitutes a much smaller proportion of the Actinobacteria seen here, but, interestingly, displays a similar distribution to *P. acnes* with consistently more seen in AD samples.

Figure 2 summarizes the data at the family level expressed in read counts, which is an indicator of bacterial numbers, not an absolute or relative measure. Figure 2A shows that the apparent raised levels of bacterial reads in AD samples are in large part, and most consistently, due to Actinobacteria; *Propionibacteriaceae*. Further BLASTN searches using representative sequences from the top three *Propionibacteriaceae* OTUs against the NCBI 16S rRNA database revealed them all to be *Propionibacterium acnes* (*P. acnes*; score: 259/259; coverage: 100%; E value: 2e-69; identity: 100%). The other major contributors are from Firmicutes with *Staphylococcaceae* as the major component, but only present in one AD sample in overwhelming numbers.

Figures 2B–G displays individually the read count data from those taxa that contribute most significantly and shows that, in addition to the marked increase in *Propionibacteriaceae* in AD samples, Actinobacteria; *Corynebacteriaceae* shows a similar pattern, although at much lower levels. Firmicutes *Staphylococcaceae* and Firmicutes “other” may also maintain this bias, but it is less clear for Proteobacteria “other”. Proteobacteria *methylobacteriacea*, in contrast, although present at relatively high levels, is fairly evenly distributed between controls and AD samples. Other taxa were deemed to have too few counts to analyze in this manner.

## Discussion

This 16S rRNA NGS study was carried out using normalized, re-amplified libraries and, although not designed to assess absolute bacterial load in samples, unexpectedly a pattern emerged with AD samples yielding noticeably more bacterial reads than controls.

### 16S rRNA Gene Sequencing from a High Genomic Background

16S rRNA gene phylotyping (Woese and Fox, 1977; Woese et al., 1985; Woese, 1987; Böttger, 1989; Pace, 1997; Clarridge, 2004), combined with NGS technologies (Tringe and Hugenholtz, 2008; Arumugam et al., 2011) has revolutionized the study of the human microbiome. Since many bacteria cannot be cultured (Stewart, 2012), NGS is generally seen as the most efficient way to attempt a comprehensive assessment of bacterial arrays (Fournier et al., 2014). In addition, it has been shown that, for very low abundance taxa within a mixed population, deep metagenomics analysis is inadequate in terms of read depth and 16S amplification and sequencing by universal 16S primers is required (Mori et al., 2014). The technical parameters of such NGS studies require PCR sensitivity, broad taxonomic coverage and the ability to differentiate well between bacterial 16S rRNA and eukaryotic 18S rRNA genes (Pace, 1997). Optimizing all three is extremely difficult to achieve because broad-spectrum bacterial 16S primers tend not to differentiate between bacterial 16S and mammalian 18S rRNA genes. Additionally, both types of sample used here require amplification of unknown, but potentially extremely low levels of bacterial targets from an overwhelming amount of human background DNA. By comparison The Human Microbiome Project Consortium Human Microbiome Project Consortium (2012) typically use samples obtained by non-invasive techniques with a human genomic component of up to 80%.

We chose here to emphasize the first two requirements at the expense of the latter, using primers likely to have a broad taxonomic spectrum and high PCR efficiency, but with a high degree of similarity to their equivalent sites on the human 18S gene. The relatively extreme PCR conditions used were the result of an attempt to reduce the 18S component; that this was not entirely successful is clearly demonstrated in the data. What cannot be demonstrated is to what extent these PCR conditions affected bacterial taxonomic coverage although, at least at the phylum level, our data agrees well with other studies and within each phylum there is a wide spectrum of taxa seen.

We have provided evidence that exogenous contaminating species were not a major component of these data and that blood is likely to be the only significant source of non brain-derived bacteria. Control brain displays similar bacterial profiles to blood whereas AD brain has a larger proportion of Actionobacteria. We propose that the levels of 16S sequences seen here in normal brains may be derived in part from its blood content and from contaminating species introduced through the NGS process. This means that these data show between 5 and 10 fold higher levels of bacterial reads in AD brain compared with control. These comparisons show that the species largely responsible for most of the increased bacterial levels seen here in AD brains, Firmicutes “other”, *Staphylococcus* and *Propionibacteriaceae* (*P. acnes*) also form the main differences between AD temporal tissue and blood, with *Propionibacteriaceae* completely absent from the blood data.

### *P. acnes* as a Possible Contributing Factor in Neuroinflammation

*P. acnes* is a commensal, gram-positive component of the human skin and mouth microflora that prefers anaerobic growth conditions and it is becoming increasingly clear that it is a significant opportunistic pathogen. Most commonly, it has been associated with post-operative lesions and implanted prostheses, but, also in chronic diseases such as lumbar region inflammation, endocarditis, sarcoidosis and in intracranial lesions (Buchanan et al., 1982; Bhatia et al., 2004; McDowell et al., 2013). Recently Branton et al. (2016) have shown the presence of Proteobacteria and Actinobacteria (containing *Propionibacteriaceae*) in both normal and multiple sclerosis affected brains; thus, normal brain has a microbiome consisting largely of Proteobacteria and Actinobacteria. Our data suggests that Actinobacteria (*P. acnes)* increases in AD brain over and above Proteobacteria. The ability of *P*.* acnes* to non-specifically stimulate the innate immune system is well documented (Tanghetti, 2013): it secretes chemotactic and proinflammatory cytokine-inducing factors and can activate complement pathways and produces hyaluronidases, proteases and neuraminidases, thought to cause epithelial permeabilization and inflammatory infiltration (Bhatia et al., 2004). It is interesting to note that *P. acnes* was cultured from three out of four biopsies from AD-affected brains (Kornhuber, 1996). *P. acnes* is a well-documented contaminant of NGS techniques as shown by no template controls and of clinical samples that have unavoidable contact with skin (Lusk, 2014; Salter et al., 2014; Mollerup et al., 2016). However, the consistently high levels seen here in AD samples compared to normal brains and the apparent minimal contribution of post mortem interval along with the lack of significant contact with skin makes contamination an unlikely explanation for the *P. acnes* content of these data. Furthermore, the physiological characteristics of *P. acnes* (Buchanan et al., 1982; Bhatia et al., 2004; McDowell et al., 2013; Tanghetti, 2013), including its known ability to grow slowly in the cortex (Kornhuber, 1996), would make *P. acnes* a good candidate for a bacterial source of neuroinflammation in AD brains and the bacterial reads seen here would warrant further investigation.

The closely related *Corynebacteriaceae* reported here are only defined to an uncharacterized culture (and not *C. diphtheriae*), but it is perhaps worth noting a report suggesting that *C. diphtheriae* is often found in the nasopharynx and that inoculation against diphtheria may provide protection against AD (Merril, 2013). Furthermore, *Corynebacteriaceae* have been detected by 16S NGS in cerebrospinal fluid from living individuals (Salzberg et al., 2016).

### AD-Associated Neuroinflammation and Possible Microbial Contributors

These data need to be viewed in the context that the mean age of the NGS cohort was 81.8 for control and 79.8 for AD. Immune response is known to be affected by age, with a waning in function of the adaptive immune system with increasing age (Weksler et al., 2005; Castelo-Branco and Soveral, 2014) whereas the innate immune system remains relatively intact, providing a rapid but short-lived acute defense against pathogens. Half of all genes upregulated in an age-related manner are associated with inflammation, oxidative stress and inflammatory cytokines (Prolla, 2002), which is consistent with evidence showing that the aging innate immune system takes on an ever-greater role against pathogens; changing from a first line of defense to a chronic, inflammatory response (Licastro et al., 2005). Consistent with this, neuroinflammation appears to be both a general age-related feature in the brain (Lynch, 2010) and, in exaggerated form, as an important contributor to many age-related neuropathological diseases (Akiyama et al., 2000a; Heneka et al., 2015a). For instance, increased levels of proinflammatory cytokines such as tumor necrosis factor alpha (TNFα) and interleukins, IL6 and IL1β are highly expressed during the early stages of AD (Sudduth et al., 2013) with levels of TNFα 25-fold higher in AD cerebrospinal fluid than controls (Tarkowski et al., 1999). Microglial cells associated with plaques in AD brains (Perlmutter and Chui, 1990) are an important component of the innate response, and are activated as a function of age (Norden and Godbout, 2013). In AD brain this is likely to be a chronic response to Aβ (Sastre et al., 2006) resulting in localized immune responses (Akiyama et al., 2000a), production of damaging free radicals and the assembly of inflammasomes which promote an escalation of neuroinflammation and neurodegeneration (Malik et al., 2015). Overproduction or reduced clearance of Aβ may exacerbate this. Additionally, the integrity of the blood-brain-barrier (BBB) diminishes, partly with age, increased cytokine load and in the context of apolipoprotein E4 (ApoE4; Bell et al., 2012).

The evidence for a significant microbial presence in the human brain is substantial, including pathogens such as fungi (including yeast; Pisa et al., 2015), herpes simplex virus-1, HIV, toxoplasma, viroids, hepatitis C, cytomegalovirus, and a variety of bacteria (Miklossy, 2011; Mawanda and Wallace, 2013; Harris and Harris, 2015; Olsen and Singhrao, 2016; Zhan et al., 2016). These could be present as the consequence of an increased permeability of the BBB. However, since Aβ42 found in plaques has anti-microbiological activity, protecting against both bacterial and fungal infections (Soscia et al., 2010; Heneka et al., 2015a; Kumar et al., 2016; Spitzer et al., 2016) their presence may be indirect evidence for a microbial-induced neuroinflammatory response. Aβ42 itself seems to be implicated as part of the innate immune response to bacterial infection. The observation that both alpha synuclein and Aβ42 have antimicrobial activity and that the CsgA (Curli) protein expressed by some bacteria (as part of their extracellular biofilm matrix) promotes alpha synuclein fibrillar deposition in the context of Parkinson’s disease (Chen et al., 2016) may suggest a role for biofilm matrix components in the propagation of host amyloid fibrillization. These observations combined with those showing age-related increase in the permeability of the gastro-intestinal epithelium (Tran and Greenwood-Van Meerveld, 2013) provide a model of aging brains under increasing threat from almost every known type of microbe, which could account for a considerable portion of age-related neuroinflammation.

### Bacteria and AD

Probably the bacteria most frequently described as associated with AD are those of the oral microbiome. Several epidemiological studies have shown links between tooth loss, poor oral hygiene and an increased risk of dementia (Gatz et al., 2006; Stein et al., 2007; Kamer et al., 2009; Paganini-Hill et al., 2012). Multiple studies have shown up to a seven-fold higher density of oral bacteria in AD brain tissues compared to normal (Miklossy and McGeer, 2016). Spirochetes such as Treponema have been linked to AD (Riviere et al., 2002) and increased levels of immunoglobulin to *P. gingivalis* (Sparks Stein et al., 2012), *F. nucleatum* and *P. intermedia*) have all been associated with cognitive impairment and/or AD (Riviere et al., 2002). *Helicobacter pylori* (Miklossy et al., 2006; Kountouras et al., 2014; Miklossy, 2015) and the spirochete *B. burgdorferi* (MacDonald and Miranda, 1987) have also been found. There is also strong evidence to suggest that the gut microbiome is associated with pathogenic mechanisms in both Parkinson’s disease (Sampson et al., 2016) and AD (Minter et al., 2016). In a mouse model of Parkinson’s disease, the gut microbiome was shown to be required for pathology as well as characteristic motor deficits (Sampson et al., 2016). Further to this, in an AD mouse model with familial AD mutations which produce numerous amyloid (Aβ) plaques in the brain, antibiotic-related changes in the gut microbiome resulted in a decrease in plaque load (Minter et al., 2016). Additionally, changes in the microbiome have been reported in obesity (Ley et al., 2005; Turnbaugh et al., 2009) and type 2 diabetes (Qin et al., 2012; Karlsson et al., 2013), each of which is strongly associated with AD.

Some of the best-documented bacterial species associated with periodontal disease were not observed in this study (Balin et al., 2008; Fujii et al., 2009; Dewhirst et al., 2010; Achermann et al., 2014). However, that these “missing” species could be present at low copy numbers or in discreet areas not sampled cannot be discounted; further sampling and NGS-based experiments exploring more rRNA gene variable regions, different PCR conditions and systematic analysis of 16S DNA in different areas of the brain are required in order to provide a fuller assessment. There are other considerations also: for instance, the cohort assessed here was not selected based on periodontal or any other disease; future studies would require specific cohorts selected for the presence and absence of periodontal or other disease. Additionally, any infection, which initiates the neuropathology of AD, may occur 15–20 years pre-mortem; therefore the bacteria identified here may be due to secondary infection after BBB breakdown. In addition, cohorts from different geographical regions may differ in their gut, mouth and brain microbiomes. Species vary between global regions and ethnic groups (Rylev and Kilian, 2008): Spanish periodontitis patients were more likely to harbor oral *P. gingivalis* than in Netherlands where *A. actinomycetemcomitans* was more evident (Sanz et al., 2000). Furthermore, whereas up to 90% of North American samples of AD brains contained *C. pneumoniae*, in another study from North European patients, *C. pneumoniae* could not be detected. (Gieffers et al., 2000). Likewise, in this study *C. pneumoniae* was completely absent, as was *E. coli* K99 (Zhan et al., 2016) and some other periodontal species previously associated with AD were noticeable by their extremely low levels or complete absence; also, some other non-oral bacterium commonly associated with AD.

## Summary

This is a novel comparative pilot study using 16S ribosomal NGS to assess the bacterial component of the microbiome in frozen and fixed post-mortem tissue from AD and control temporal cortex. The study presented here has shown, for the first time, that 16S NGS in terms of both PCR sensitivity and taxonomic coverage is extremely well suited to the detection and analysis of bacterial populations in both frozen and FFPE temporal cortex, despite background human genomic DNA being present in overwhelming excess. Although this is only a pilot study with a limited cohort, these data strongly suggest that AD brains tend to have strikingly large bacterial loads compared to controls. In this study, species associated with skin, nasopharyngeal and oral areas such as Firmicutes and most consistently Actinobacteria, especially *P. acnes* (up to 94% of Actinobacteria) are responsible for this.

## Author Contributions

DCE, SJA, DKS devised the concept in collaboration with TEB, NXW and MD. DCE, with DKS and TLC conducted experimental work on tissue extraction and PCR analysis. CMW and JAC performed the NGS process; TEB processed NGS data; DCE interpreted NGS data. DCE and SJA, with DKS drafted the manuscript with assistance from all other authors. All authors critically revised the article and approved publication.

## Conflict of Interest Statement

The authors declare that the research was conducted in the absence of any commercial or financial relationships that could be construed as a potential conflict of interest.

## Footnotes

^1^http://qiime.org/

## Supplementary Material

FIGURE S1 Gel electrophoresis analysis of amplicons. Using an overlapping but extended cohort of frozen samples to that used for the next generation sequencing (NGS) study, amplicons were analyzed by higher resolution gel electrophoresis.
